# An Experimental Study of Volatile Organic Compound (VOC) Emissions from a Resin 3D Printer to Assess Exposure and Exposure Mitigation

**DOI:** 10.1021/acs.chas.5c00167

**Published:** 2025-12-17

**Authors:** Danielle A. Baguley, Delphine Bard, Gareth S. Evans, Paul S. Monks, Rebecca L. Cordell

**Affiliations:** † School of Chemistry, 4488 University of Leicester, University Road, Leicester LE1 7RH, U.K.; ‡ University of Northumbria, College Street, Newcastle Upon Tyne NE1 8ST, U.K.; § 4933 Health and Safety Executive (HSE), Science Division, Harpur Hill, Buxton SK17 9JN, U.K.; ∥ Insight for Work, Sheffield S17 3PD, U.K.

**Keywords:** volatile organic compounds, 3D printing, resin printing, exposure assessment, indoor air quality, emission mitigation, vat photopolymerization

## Abstract

Recent increases in the popularity of affordable 3D printers necessitate research to investigate the potential volatile organic compound (VOC) exposure that an operator would experience. VOC emissions from a Formlabs Form 2 were tested using four different resins (Clear, White, Tough, and Elastic) across several time-resolved tests and exposure scenarios: an enclosed test chamber, and within a ventilated room at two distances, with an extraction hood to investigate “real-world” exposure scenarios and the impact of mitigation methods. 2-Hydroxypropyl methacrylate, 3-hydroxypropyl methacrylate, and 2-hydroxyethyl methacrylate were the prominent VOCs emitted from the resin 3D printing process, among other acrylic-based compounds. The composition of the VOCs was dependent on the type of resin: Elastic resin emitted a greater diversity of compounds, including the previously unreported isobornyl acrylate, while Tough resin emitted higher concentrations of smaller cross-linking compounds such as 2-hydroxyethyl methacrylate. VOC emissions peaked at the end of the active printing process when the build plate rose from the liquid resin bed. In the enclosed chamber scenario, total VOC (TVOC) concentrations exceeded 128,000 μg/m^3^, representing worst-case poorly ventilated conditions. Under realistic room conditions, TVOC concentrations reached 45–116 μg/m^3^ at 50 cm from the printer and returned to baseline within 2 h after printing ended. The TVOC emission concentrations were significantly reduced by 71–84% when the distance between the printer and the sampling position was increased from 0.5 to 2 m, or when an extraction hood fitted with a carbon VOC filter and particulate HEPA filter was used. These two exposure mitigation methods were considered practical options for home users, “maker” communities, and schools to use. While individual VOC concentrations remained well below established workplace exposure limits, many identified compounds lack published safety guidelines, making health risk assessment challenging, and both their acute and chronic health impacts remain unknown.

## Graphical abstract

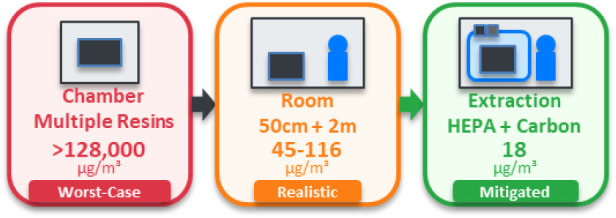

## 1 Introduction

3D printing, a subtype of additive manufacturing, has been a growing field since the method was devised in the 1980s and has grown in use over the last two decades.^1,2^ Specifically, it has been gaining use in industries such as medical devices^3,4^ and dentistry, as well as in spaces such as schools, home use, businesses, and even libraries. The 3D printing technique adds layer by layer of different materials to create a three-dimensional structure of a product. Vat photopolymerization is one of the seven main subtypes of 3D printing and employs a light source to selectively cure photopolymerizable resins in a resin bed, forming the desired shape.^5–12^ During the build process, 3D printers release volatile organic compounds (VOCs) and particles into the air that may be inhaled. The composition of the emitted compound mixtures changes depending on the type of printer and the materials used.

There is currently no published guidance for consumers on how to use 3D printers safely. In contrast, such guidance has been published in the UK education sector,^13,14^ and the American research agency National Institute for Occupational Safety and Health (NIOSH) gives recommendations on “exposure control” guideline values for educational establishments, makerspaces, libraries, and small businesses. Given the widespread public use of 3D printing technologies, investigating their safety and potential longer-term health effects is a priority. Indeed, there is a broad range of potential hazards associated with their use, including burns, inflammation, irritancy, and allergic reactions that affect both the skin and the respiratory tract.^15,16^

The liquid resins used in some 3D printers contain a mixture of volatile chemicals (e.g., solvents) and less volatile chemicals (e.g., polymers). Only some of these substances are classified as hazardous under EU and UK chemical regulations. A mixture will only be classified as hazardous if one or more constituents are classified as hazardous above a certain threshold. An unresolved concern is whether a mixture of VOC emissions from resin bed printers could cause adverse health effects that cannot be predicted by considering the individual chemical constituents of this mixture.

A commonly used type of 3D printing, material extrusion, including the popular fused deposition modeling (FDM), has been researched more thoroughly^8,17–34^ compared to liquid resin 3D printers. In contrast, there is relatively limited literature quantifying VOC emissions from vat photopolymerization.^6,9–11,34–53^ A recent review assessed the known evidence about VOC emission data. Four published studies identified and quantified a range of VOCs from 3D printing processes using resins supplied by Formlabs.^34,38,44,48^ Of these VOCs, isopropanol was quantified with the highest concentration of 1374 μg/m^3^, acetone at 581 μg/m^3^, and 2-hydroxypropyl methacrylate at concentrations of up to 21.9 μg/m^3^. The other identified compounds were mainly acrylic, carbonyl, or alkene class compounds. Acrylic compounds comprise a significant percentage of the liquid resin products as these are catalytically polymerized into the final resin model using a photoinitiator catalyst.^5,9,54^

Some of the identified constituents of these resins, including formaldehyde, acetaldehyde, acetone, and styrene,^34,38,44,48^ are classified as potentially hazardous to human health. Acute exposure to VOCs can potentially lead to respiratory irritancy, particularly in vulnerable groups such as the elderly, children, and those with preexisting health conditions.^55,56^ Long term exposure to VOCs can lead to more chronic health outcomes.

To protect the members of the public exposed to VOCs in outdoor and indoor environments, international bodies such as the World Health Organization (WHO) have identified air quality guidance values. National bodies such as the UK-HSA (United Kingdom Health Security Agency) are also responsible for setting specific guidance values for air quality, including hazardous VOCs. These values are published to improve air quality and reduce the risks to health. In the workplace, to assess whether exposure is below the occupational exposure limits, known as workplace exposure limits (WELs) in the United Kingdom (UK), air sampling is typically conducted in the breathing zone of the worker.

The adverse health effects of VOC exposure are directly linked to the concentration and duration of the exposure. By reducing the concentration and the duration of exposure, the adverse effects of VOCs can be mitigated using air purifiers, room air filters,^11,47,57^ and increased ventilation. Reducing emissions at the source is the most effective preventive measure.

Previous investigators concluded that multiple VOCs are emitted from 3D printers, depending on the type of printers and materials used, though mainly focusing on plastic filament-based 3D printers. This research aims to investigate the VOC emission profiles from a resin bed 3D desktop printer using liquid resin of different compositions under different simulation scenarios of an enclosed test chamber and an unoccupied room setting. These scenarios were chosen to establish whether these emissions could reach concentrations potentially harmful to an operator and whether they could be reduced at the source using practical control measures.

## 2 Methodology

### 2.1 Materials

A high-quality desktop resin 3D printer (Formlabs, Form 2) was used to simulate emission scenarios in a test chamber and room setting. This is a type of small 3D printer that produces excellent-quality resin-based printed structures. It is fitted with a nonventilated UV protective hood surrounding the liquid resin bed to prevent unwanted resin curing in ambient light. The hood was not airtight or ventilated. Previous research has also used Formlabs printers for VOC emission research.^34,38,44,48^

The tested Formlabs resins were named Clear (FLGPCL04), Elastic (50A), Tough (FLTOTL05), and White (FLGPWH04), all purchased in 2022. The exact composition of each resin is proprietary and has not been released by the manufacturer. The chemical constituents are named in the safety data sheets, and the predominant constituents are urethane dimethacrylate and methacrylate monomers, as well as a photoinitiator. The Elastic and Tough resins are examples of different specialized functions of the printed structure, while the Clear and White resins are examples of differently colored resins.

### 2.2 Scenarios and Sampling

Air samples were collected using conditioned sorbent tubes packed with Tenax TA/Carbograph 1TD (Markes International) for VOCs analysis using thermal desorption gas chromatography–mass spectrometry methods. Three scenarios were investigated, and three repeats were conducted for each of the test scenarios and resin types. Owing to the wide dynamic range of concentrations observed during this study, the sampling flow rate and duration differed depending on the test scenario. The temperatures within the test chamber and test room were ambient within the research building. These varied seasonally between 19 and 22 °C. All repeats of each type of resin were performed within 2 weeks.

### 2.3 Test Chamber Scenario

The 3D printer was placed inside a test chamber adapted from a fume hood (Monmouth Scientific, Special Circulaire 900) along with a CO_2_ monitor (Rotronic, Northants). The test chamber was made with acrylic sides, a metal back, and a metal work surface. The layout of the hood is given as a bird’s eye view in Figure 1. There was no circulating air mechanism included, though the air inlet was low on the right-hand wall and the sampling port was high on the left-hand wall, allowing air to pass over the 3D printer before sampling. The chamber had a volume of 0.74 m^3^ and an air turnover rate of 0.3 h^–1^. The VOCs emitted from the different resins during the printing process and immediately after printing ended were characterized. Compressed air was delivered to the chamber at a rate of 4 lpm (BTCA 178 grade, BOC, UK), resulting in an air exchange rate of 0.3 h^–1^ (240 L per hour within a 0.74 m^3^ hood), which is representative of poor dwelling ventilation rates. This controlled chamber design served dual purposes: to characterize source emission profiles and temporal patterns without confounding dilution effects, and to simulate worst-case conditions representative of small, poorly ventilated spaces (e.g., closets, small workshops) where users might place printers without adequate ventilation awareness. The composition of the compressed air was 19.25% oxygen with nitrogen and minimal levels of carbon monoxide (<1 ppm), carbon dioxide (<300 ppm), NO_*x*_ (<0.1 ppm), and total hydrocarbons (<0.1 ppm). The schematic of the adapted particle hood environment is shown in Figure 1.

**Figure 1:**
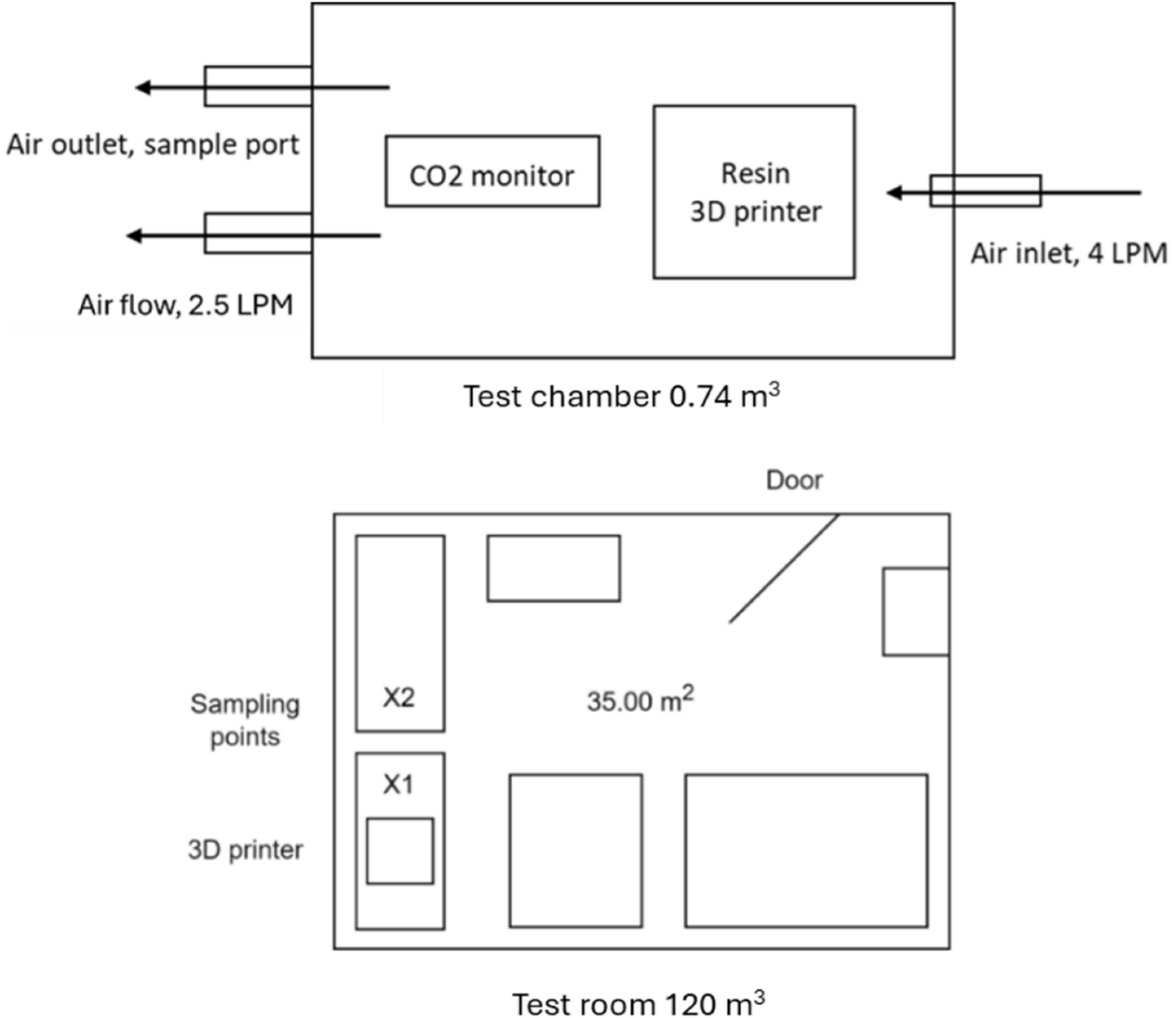
Schematic of the test chamber (upper) setup and schematic of the experimental room (lower). The sampling ports within the chamber have been included, along with the associated flow rates. The position of the 3D printer and the two sampling locations within the room are shown, X1 and X2.

For each sample, 100 mL of air was collected in a conditioned sorbent tube using an Acti-VOC Plus pump (Markes International) operating at 100 mL/min for 1 min.

Three samples were collected when the test chamber was sealed, 15 min before printing, and a sample was taken when the printer started to print (time = 0 min). Additional samples were taken during printing. The print time using the Elastic and Tough resins was considerably longer, and samples were collected every 30 min. White and Clear resin printing had a shorter print time; samples were collected every 20 min. All resins tested had samples collected after printing and at 0 (as the build plate rose), 15, 30, 45, and 60 min (Table 1). The sampling position port is shown in Figure 1.

**Table 1:** The Sampling Conditions during the Chamber exposure Scenarios^a^

Time of samples	Activity sampled	Sample numbers
–15 min, before print starts	Printer turned off, cold. Baseline in the chamber	1–3
–10 min, before print starts	Printer warming to print temperature, chamber closed for the final time	
0	Printing begins	4
0–125 min (resin type dependent)	Active printing of dice design. Samples every 20–30 min	5–9
End of print −60 min after print ends	Printing ended, cooling down to room temperature, with continued air flow. Samples taken every 15 min	10–14

a All samples were 1 min in length.

#### 2.3.1 Test Room Scenario 1 (Printer Not Enclosed)

The 3D printer was set up in a room with a fresh air supply rate of approximately 10 L/s/person capacity (approximately 1.02 room air exchanges per hour) to simulate more realistic user exposure scenarios where a 3D printer may be set up (e.g., garage or hobbyist environment). The same Clear resin investigated in the chamber scenario was used for all of the test room scenarios. The room was approximately 120 m^3^ with tables set in the space. The 3D printer was placed on a table in one corner of the room with sampling distances set at 50 cm and 2 m (X1 and X2, Figure 1). VOC samplers were placed around the 3D printer, where an individual might sit adjacent to the printer, which was placed on a table. The experimental operator turned on the printer and left the room for the sampling duration.

#### 2.3.2 Test Room Scenario 2 (Printer Enclosed)

Test scenario 2 involved the same experimental room and setup as test room scenario 1, except that the 3D printer was encased within a 3D printer safety hood (SC-01, KORA). This hood extracted air through a HEPA filter and an activated carbon filter before releasing it into the room environment. The samples were placed directly next to the 3D printer inside the extraction hood and taken at the same 50 cm position as in scenario 1.

For the two test room scenarios, 1 L of air was collected in each conditioned sorbent tube using an Acti-VOC Plus pump operating at 50 mL/min for 20 min. An autosampler was used to change tubes every 20 min. For each experiment, 14 samples were taken (Table 2). The sampling period took 4 h and 40 min. The VOC sample 1 (0–20 min) was collected while the 3D printer was disconnected and served as a baseline measurement in the room with the cool, inactive 3D printer. The 3D printer was turned on, and a print was programmed at the 20 min mark. Before printing, it warmed up for about 20 min to a temperature of 31 °C, and the print began around the 40 min sampling mark. The printing continued for 2 h and 5 min, and samples were collected for 2 h after the printing ended to track the decay in VOC concentration. The sequence of events is illustrated in Table 2.

**Table 2:** The Sampling Conditions during the Test Room Scenarios

Time of samples	Activity sampled	Sample numbers
0–20 min	Printer turned off, cold, baseline in room	1
20–40 min	Printer warming to print temperature	2
40 min–2 h 40 min	Active printing of dice design	3–8
2 h 40 min–4 h 40 min	Printing ended, cooling down to room temperature	9–14

### 2.4 Sample Preparation and Analysis

The samples were analyzed using a Unity-2 thermal desorption unit (Markes International, Cardiff, UK) connected to a gas chromatography instrument (Agilent 7820A), which was coupled to a single quadrupole mass spectrometer (Agilent 5977B).

Before analysis, each tube was manually spiked with 1 μL of an internal standard solution (totaling 11.88 ng chlorobenzene-*d*_5_, 11.88 ng bromochloromethane, 11.88 ng 1,4-difluorobenzene, and 17.70 ng 1-bromo-4-fluorobenzene, all from Restek, UK). The 1 μL solution was injected into a 100 mL/min flow of purified nitrogen for 120 s. During analysis, chlorobenzene-*d*_5_ was used as the internal standard.

Owing to the high dynamic range of VOC concentrations, two TD-GC-MS methods were required to analyze the samples.

For the test chamber scenario, the tubes were desorbed after sampling at 300 °C for 5 min with a flow rate of 45 mL/min and a split flow rate of 25 mL/min. The volatile compounds were loaded onto a “hydrophobic, general-purpose” trap, which was cooled and held at −10 °C. The trap was then heated at 300 °C for 5 min with a flow rate of 20 mL/min and a split flow rate of 100 mL/min before entering the transfer line to the GC column (Rtx-Wax column, 30 m × 0.25 mm ID × 0.25 μm df). Each sample was split in two places, first as it traveled from the tube to the trap (1.6:1 split) and second from the trap to the transfer line (51.0:1 split), totaling an overall split of 79.3:1.

For the analysis, helium (N6.0 grade, BOC) was used as the carrier gas at a flow rate of 2 mL/min. The temperature within the GC-MS oven started at 35 °C, increasing by 5 °C per minute to the final hold temperature of 250 °C, where it was held for 10 min. The total GC-MS run time was 53 min. The mass spectrometer had an electron ionization source (70 eV) and a single quadrupole mass filter with a scan range of 40–450 *m*/*z* and a frequency of 3 Hz.

For the test room scenarios (both enclosed and not enclosed), the sample tubes were desorbed at 300 °C for 5 min with a flow rate of 45 mL/min without a split. The volatile compounds were collected onto the same “hydrophobic, general-purpose” trap, cooled, and held at −10 °C. The trap was heated at 300 °C for 5 min with a flow rate of 20 mL/min before entering the transfer line to the same GC column as before (Rtx-Wax column, 30 m × 0.25 mm ID × 0.25 μm df).

For the analysis of samples from all chamber and room test scenarios, helium (N6.0 grade, BOC) was used as the carrier gas at a flow rate of 2 mL/min. The temperature within the GC oven started at 35 °C, increasing by 5 °C per minute up to the final hold temperature of 250 °C, where it was held for 10 min. The total GC-MS run time was 53 min. The mass spectrometer had an electron ionization source (70 eV) and a single quadrupole mass filter with a scan range of 40–450 *m*/*z* and a frequency of 3 Hz.

### 2.5 Data Analysis

The data were analyzed using MassHunter Agilent software, using MassHunter Unknowns to identify peaks within the chromatogram with at least 50% match score and consistent mass spectral matching using the Qualitative analysis package. The quantification of the compounds was achieved using the Quantitative package and the library populated from Unknowns peak picking. Chromatographic peaks were identified using 50 and 100 window deconvolutions with 100,000 absolute area and library matching to the National Institute of Standards and Technology library (NIST 2011)^57^ or using reference standards purchased from ThermoFisher. Compounds that were identified using standards are reported in Table 4 as concentrations, while compounds that were identified only by their mass spectral data matched in NIST were reported as being present in the samples without quantification.

Each compound was quantified using an internal standard and a unique calibration curve (Table 3). The compound peaks from the scenario and calibration samples were integrated and represented as a ratio to the internal standard peak (chlorobenzene-*d*_5_). Since both the calibration standards and the samples use the same ratio-based representation, the measured ratios from the samples can be compared directly to the calibration curve to determine the concentration of each compound.

**Table 3:** The Calibration Concentration Range for VOCs That Were Quantified from Both the Chamber and Room Exposure Scenarios^a^

VOC	Chamber exposure concentrations, ng	Room exposure concentrations, ng
1-Hexanol, 2-ethyl	100–500	3–100
2-Hydroxyethyl methacrylate (2-HEMA)	1000–5000	6–200
2-Propenoic acid	100–500	3–100
2-Propenoic acid, 2-methyl	100–500	3–100
2-Hydroxypropyl methacrylate (2-HPMA)	540–10,800	3–108
3-Hydroxypropyl methacrylate (3-HPMA)	460–9200	2–92
Camphene	100–500	3–100
Ethanol, 2-butoxy	100–500	3–100
Isobornyl acrylate	100–500	3–100
Methacrylic acid, methyl ester	100–500	3.13–100

a During experimentation, 100 mL samples were taken from the chamber due to the high concentrations observed, though they have been reported as per liter. The room air was quantified per liter. All VOC amounts added for calibration are given in ng added .

### 2.6 Statistical Analysis

Statistical analysis was undertaken using SPSS v28 (Statistical Package for the Social Sciences). A Kolmogorov–Smirnov test was performed to assess the homogeneity of variance in the data set and the normality of the data within each resin or distance type. A parametric two-way ANOVA was performed using SPSS to identify significant differences between data sets of the comparable resins. A Tukey HSD post hoc test was performed to compare the mean concentrations of VOCs, especially between the White and Clear resins and the Tough and Elastic resins.

## 3 Results and Discussions

### 3.1 Results for the Test Chamber Scenario

There was a wide dynamic range in the concentrations of the VOCs in the samples analyzed. The VOCs with the highest concentrations from the chamber scenario were analyzed separately from the rest using a high-splitting method. Three compounds, 2-hydroxypropyl methacrylate (2-HPMA), 3-hydroxypropyl methacrylate (3-HPMA), and 2-hydroxyethyl methacrylate (2-HEMA), were found in this high concentration range. They formed overlapping peaks, which were effectively separated into distinct individual peaks using the split analysis method.

2- and 3-Hydroxypropyl methacrylate and 2-hydroxyethyl methacrylate were the VOCs with the highest concentrations measured from Clear, White, and Tough resins (Table 4). The Elastic resin emitted these VOCs in lower concentrations (Table 4, Figure 2). The lower 2-HEMA and HPMA emissions from the Elastic resin are potentially a consequence of the increased number of VOCs released from this resin. This may be due to the lower concentration of the HEMA and HPMA compounds in the liquid resin itself due to the additional compounds within the mixture. This would then translate to lower concentrations within the liquid resin, and therefore to lower emission concentrations. For example, isobornyl acrylate was probably added to the liquid resin to improve the elastic properties of the resin. In contrast, the emission concentration of 2-HEMA and HPMA was considerably lower in the Elastic resin, potentially to compensate for the additional compounds. The Tough resin had the highest quantified emissions of 2-hydroxyethyl methacrylate. The higher concentrations of this smaller molecule may support cross-linkage between polymer chains, leading to enhanced strength of the polymer structure of this resin. The Elastic resin included additional constituents such as nonpolymerizable compounds, which may reduce the polymerization and increase the flexibility of the final product. The isobornyl acrylate would also reduce the polymerization process due to the steric bulk of the isobornyl group in the molecule, leading to a more flexible structure.

**Table 4:** Chamber Exposure Scenario—Mean Concentration from the Maxima Concentration Timepoint (μg/m^3^) of the VOCs from the Four Resins Tested, and the Identification Level^a^ Associated with Each Compound^b^

VOC	Clear	Tough	White	Elastic	MSI identification level^61^
2-Ethylhexan-1-ol	124	302	289	403	1
2-Hydroxyethyl methacrylate (2-HEMA)	2020	3890	1670	564	1
2-Propenoic acid				2500	1
2-Methyl-2-propenoic acid	122	222	250	159	1
2-Hydroxypropyl methacrylate (2-HPMA)	58,100	56,600	68,500	15,700	1
3-Hydroxypropyl methacrylate (3-HPMA)	50,600	44,300	56,800	12,700	1
Camphene				9000	1
2-Butoxy ethanol	209	1280	1290	2700	1
Isobornyl acrylate				12,800	1
Methacrylic acid, ethyl ester	Yes			268	1
2-Pentenoic acid 2-methyl		Yes	Yes		2
2-Propanol, 1-(1-methylethoxy)	Yes	Yes	Yes		2
2-Propanone, 1-hydroxy		Yes			2
2-Carene				Yes	1
3-Butenoic acid	Yes				2
4-Hydroxybutyl acrylate				Yes	2
Benzaldehyde			Yes		1
Cyclohexanone, 3,5-dimethyl-	Yes				2
Diethylene glycol hexyl ether		Yes			2
Linalool				Yes	2
Methyl methacrylate	Yes				1
*n*-Heptyl acrylate		Yes			2
Toluene				Yes	2

a Identification level denotes the Metabolomics Standards Initiative (MSI) level of identification.^61^ Level 1 identification involves matching retention time and mass spectral data against a purchased standard. Level 2 identification involves a mass spectral library match.

b The table is split to show the quantified compounds first (3sf) and then the compounds which were identified and not quantified. Compounds that were identified through a NIST mass spectral match were not quantified and are reported as being present by a “yes” indication.

**Figure 2:**
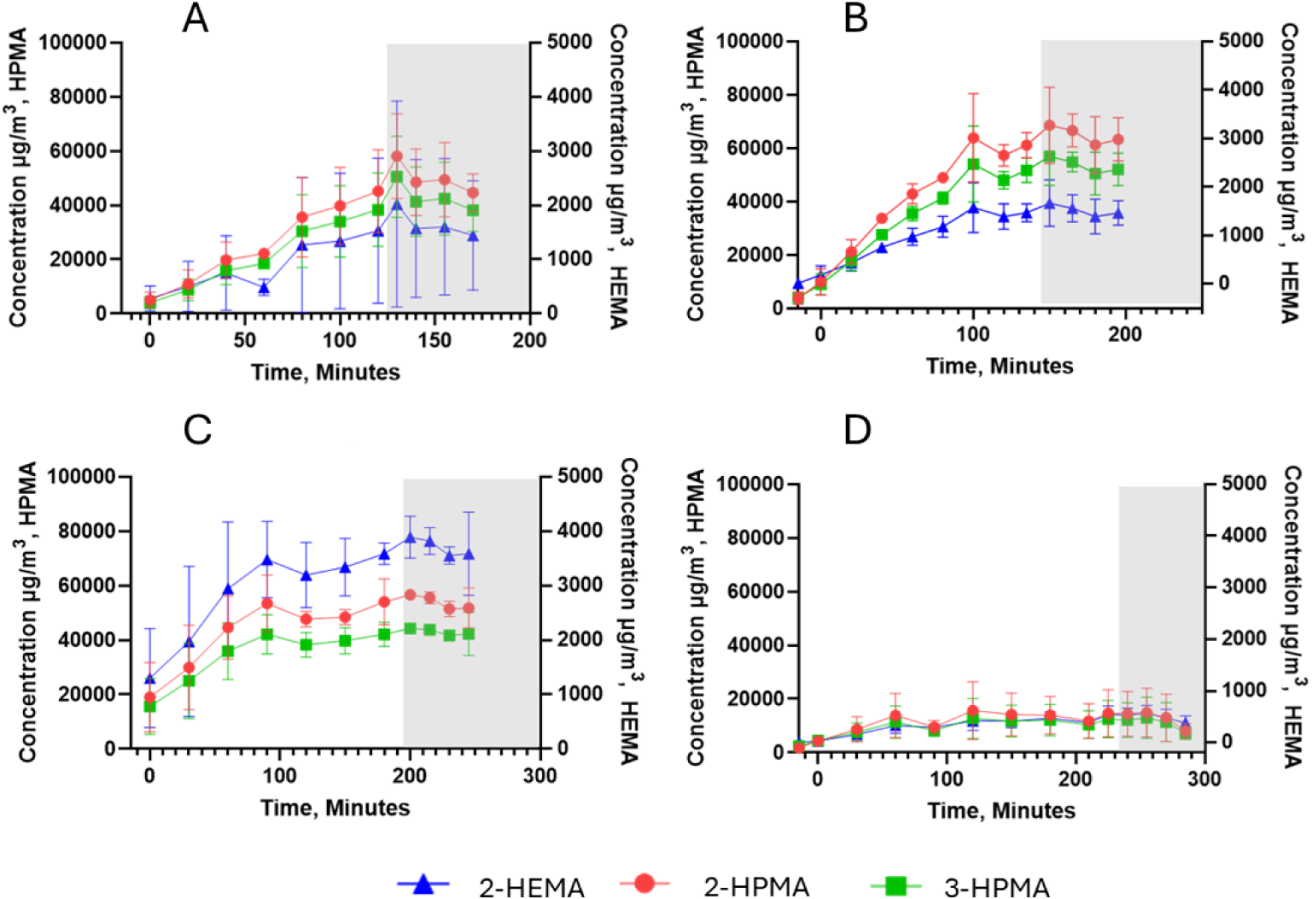
Test chamber scenario, emission profiles of 2-hydroxyethyl methacrylate (2-HEMA), 2- and 3-hydroxypropyl methacrylate (2- and 3-HPMA) concentrations (points are the mean ± error bars showing standard deviation). The gray section indicates the sampling after printing ended. (A) Clean resin, (B) White resin, (C) Tough resin, and (D) Elastic resin.

2-HEMA and HPMA have similar structures with a polymerizable acrylic group, a short hydrocarbon chain, and a polar hydroxy group. However, the isobornyl acrylate molecule has an acrylic functionality attached to a bulky isobornyl group. This difference in 3D structure may increase the flexibility of the resin owing to the added disruption between polymer chains from the large isobornyl molecules. This isobornyl acrylate group is present only in the flexible resin, further strengthening the argument that the resin base component fingerprint depends on the resin products’ functionality. The addition of camphene to Elastic resin may enhance flexibility by acting as a pore-forming agent, creating a porous structure that exhibits decreased stiffness and improved flexibility, though this has not been researched within liquid resin 3D printing, to the authors’ knowledge. Camphene and 3-carene are types of terpenes that have been linked to 3D printing through polyamide chemistry.^58,59^

Some compounds identified during this study have been identified in previous research. 2-Hydroxyethyl methacrylate has been quantified from the print process by Zhang et al.^48^ and Stefaniak et al.,^50^ and 2-hydroxypropyl methacrylate has been quantified by Zhang et al.,^48^ Stefaniak et al.,^50^ Bowers et al.,^38^ and Väisänen et al.^44^ Miller-Schulze et al. have identified many acrylic compounds emitted from resin 3D printers including 2-hydroxyethyl acrylate.^36^ In addition, many of the compounds identified in Table 4 have not been identified in previous research.^60^ Acrylic compounds, 3-hydroxypropyl methacrylate, which is the structural isomer of 2-hydroxypropyl methacrylate, as well as isobornyl acrylate, 4-hydroxybutyl acrylate, and 2-propenoic acid–based compounds, have been identified from VP resin 3D printing for the first time.

Some compounds that had been identified in previous research were not observed here.^34^ Formaldehyde was not identified in this study as the methodology was not designed to target compounds with low molecular weight or those that are very volatile, and no additional tests were carried out to target it, though it was identified by Vasilescu.^42^ In addition, acetone was not identified during the active printing process. This compound can be used as a cleaning agent, which occurs after the printing cycle ends. Sampling was not continued for the postprocessing steps, as these were often carried out multiple days after the printing of the item.

The evolution profile of the VOCs during the printing process in the test chamber was partially dependent on the resin tested. The same overall emission profile was observed for each resin. However, the peak concentrations differed. Additional compounds were also identified for the specialized function Tough and Elastic resins, shown in Figure 3.

**Figure 3:**
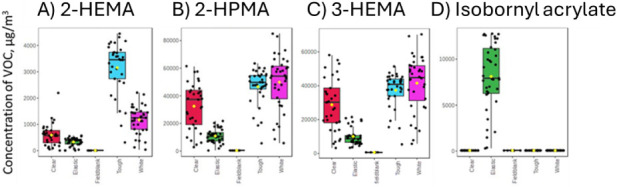
Test chamber scenario—boxplots showing both the mean and interquartile ranges of concentrations of A) 2-hydroxyethyl methacrylate (2-HEMA), B) 2-hydroxypropyl methacrylate (2-HPMA), C) 3-hydroxypropyl methacrylate (3-HPMA), and D) isobornyl acrylate.

VOC emissions increased rapidly following the commencement of printing and then plateaued at 100–160 min (Figure 2), depending on the resin. This temporal trend was observed for all the compounds in every resin. The observed time dependence is probably caused by the printer’s heating cycle, as the resin heats to a specific temperature before staying at that temperature during the entire print cycle, resulting in a constant release of VOCs. The slight increase following the plateau period was correlated with the end of printing and the build plate rising out of the resin bed. The end-of-print behavior has been observed in previous research and is thought to be due to the increased surface area on the build plate and volatilization of resin constituents into the air from the plate.^39,47^

The concentrations of the four most abundant VOCs (2-hydroxyethyl methacrylate, 2- and 3-hydroxypropyl methacrylate, and isobornyl methacrylate) are shown in Figure 3 for each resin type. Isobornyl acrylate was only released from the Elastic resin, where the mean concentration during sampling was ∼7000 μg/m^3^. During the sampling period, 2-HEMA had the highest mean concentrations in Tough resin (∼3500 μg/m^3^), then White resin (∼1500 μg/m^3^), followed by Clear resin (∼800 μg/m^3^), and Elastic resin (∼500 μg/m^3^). HPMA compounds were observed in high concentrations for Tough and White resins (∼55,000 μg/m^3^), followed by Clear resin (∼38,000 μg/m^3^) and the lowest concentration was observed for Elastic resin (∼10,000 μg/m^3^).

The test chamber had a small internal volume and maintained an air exchange rate of 0.3 per hour throughout the sampling process, including postprinting. The chamber experiments were used to identify the VOCs emitted from the print cycle and to understand the emission profiles throughout the printing process and between resin types. The concentrations of the VOCs inside the chamber would have been expected to decrease within the hour after the print process ended, as the continual air exchange would have replaced 30% of the air in the chamber. However, this effect would have been seen if the printer had no longer acted as a chemical source. The VOC concentrations did not decrease during the postprinting sampling period, indicating that the printer was still emitting chemicals.

During printing, the CO_2_ concentrations in the chamber decreased, indicating air exchange and CO_2_ displacement, as the compressed air cylinder did not contain CO_2_. The example showing the CO_2_ and temperature during the Elastic printing cycle is shown in Figure 4.

**Figure 4:**
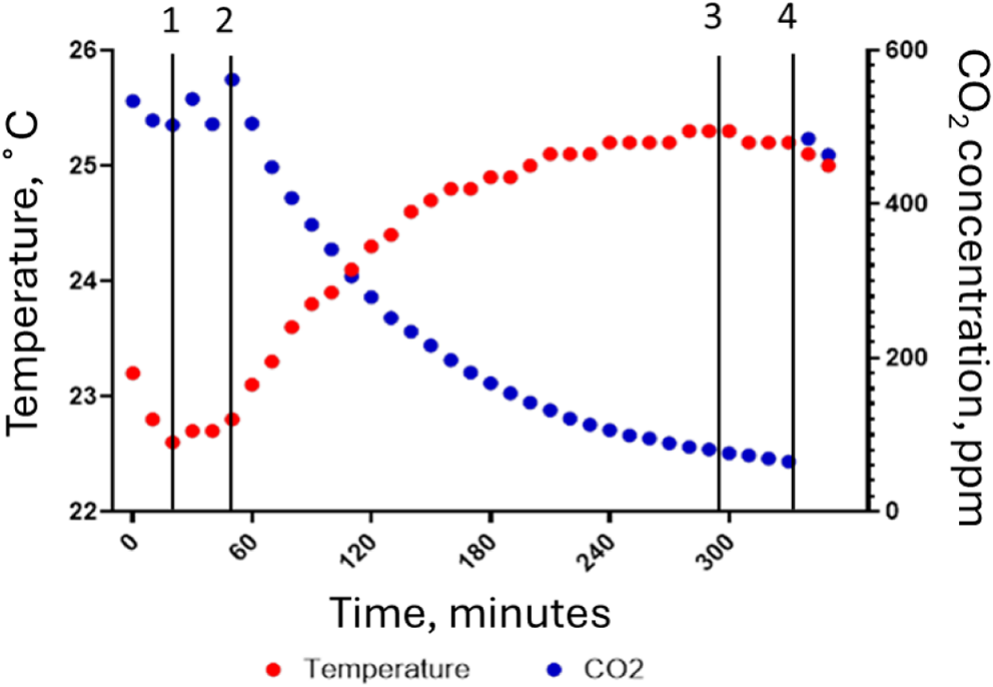
Test chamber scenario—changes in the CO_2_ concentration and temperature inside the chamber for the Elastic resin during sampling. 1 indicates when the printer was turned on; 2 indicates when the print was set to start, and the warmup began; 3 indicates when the print ended; 4 indicates when the chamber was opened after the sampling ended.

The continued emission after the printing cycle ended may have been due to the build plate. Once raised out of the resin bed, the build plate has a large surface area for the volatilization of the resin constituents, which retain heat after the printing stage, eventually cooling to room temperature. The temperature inside the chamber also decreased after the printing ended (Figure 4). The chamber scenario was designed to represent poorly ventilated conditions and to provide a controlled source characterization. Under these conditions, the observed concentrations establish worst-case scenarios for using a 3D printer in small, inadequately ventilated spaces where users might place printers. The controlled environment enabled resin-specific VOC fingerprinting and time-resolved emission profiling without the rapid dilution that occurs in larger spaces. In addition, the 3D printer had a cover surrounding the resin, which limited air exchange between the printer and the chamber. During printing, the concentration of VOCs increased in the UV hood and slowly diffused in the chamber. This maintained high VOC levels in the chamber for the 1 h of sampling after printing ended.

Concentrations of VOCs may remain high for several hours after printing, which could raise the risk of exposure in small, poorly ventilated spaces that a user might not expect.

### 3.2 Test Room Results—Unenclosed and Enclosed Scenarios

The potential exposure to a printer operator was investigated within a room environment at 0.5 and 2 m distances from the printer, with and without an extraction hood equipped with a HEPA and activated carbon filter. These tests examined the impact of distance and source extraction on practical mitigation measures. The VOC concentrations observed at 0.5, 2, and 0.5 m with an extraction hood are detailed in Figure 5, and large decreases in VOC concentration were observed at longer distances or when an extraction hood was used. The highest quantified TVOC for this test scenario was 43, 11, and 8 μg/m^3^ for scenarios 0.5 m, 2 m, and 0.5 m enclosed, respectively. The calculated TVOC for the peak emission time point using all quantified compounds and associated standard deviation for each of the three conditions are as follows: 50 cm—116.3 ± 14.1 μg/m^3^, 2 m—34.1 ± 11.2 μg/m^3^, Extraction—18.1 ± 1.2 μg/m^3^. These TVOC measurements for the quantified VOCs show the difference between the baseline scenario of having a 3D printer next to an operator and the use of either of the mitigation methods tested. These results show the impact of the distance and extraction on the TVOC concentration observed. Increasing the distance reduced TVOC from 116.3 to 34.1 μg/m^3^, a reduction of 71%. The use of extraction reduced TVOC from 116.3 to 18.1 μg/m^3^, a reduction of 84%.

**Figure 5:**
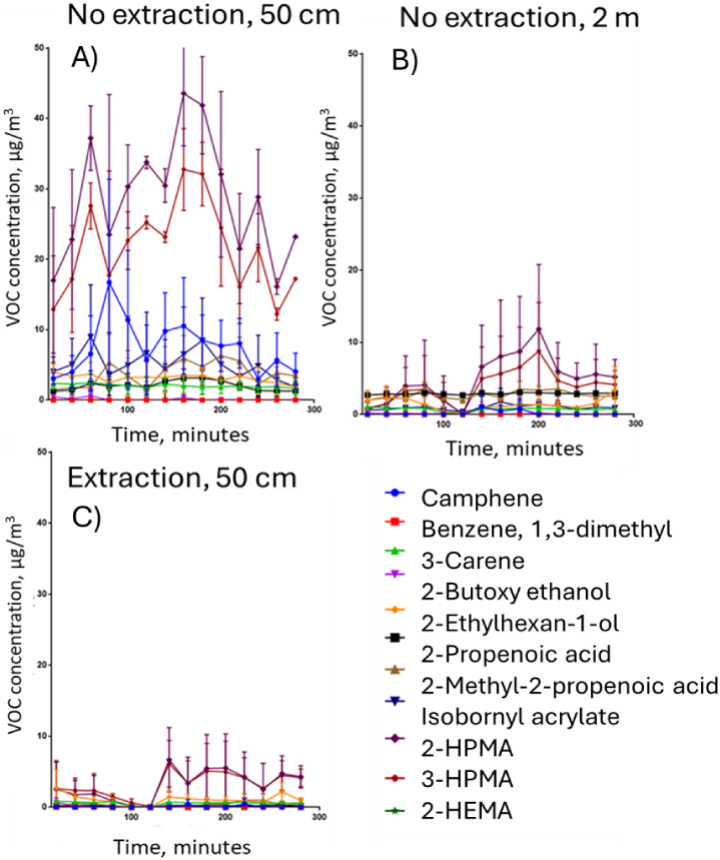
Test room scenarios: mean and SD VOC concentrations throughout the print process within the room scenario. Three scenarios were tested: (A) 50 cm from the 3D printer, (B) 2 m from the printer, and (C) 50 cm from the printer in the extraction hood.

In the unenclosed and enclosed scenarios of the test room study, the concentration of VOCs returned to the baseline before printing started, within 2 h after the printing process ended. Figure 5 shows the baseline before printing at time = 0 min, the end of printing at 160 min, and the postprinting stage, which finished at 280 min.

Exposure to individual VOCs is not the only concern. The total VOC in the air (TVOC), i.e., cumulative impact, was also assessed (Figure 6). The TVOC was calculated by adding the concentrations of each quantified VOC at each time point. Figure 6 displays the stacked concentrations of each VOC and the cumulative concentration (TVOC) at each time point at the top of the stacked graph.

**Figure 6:**
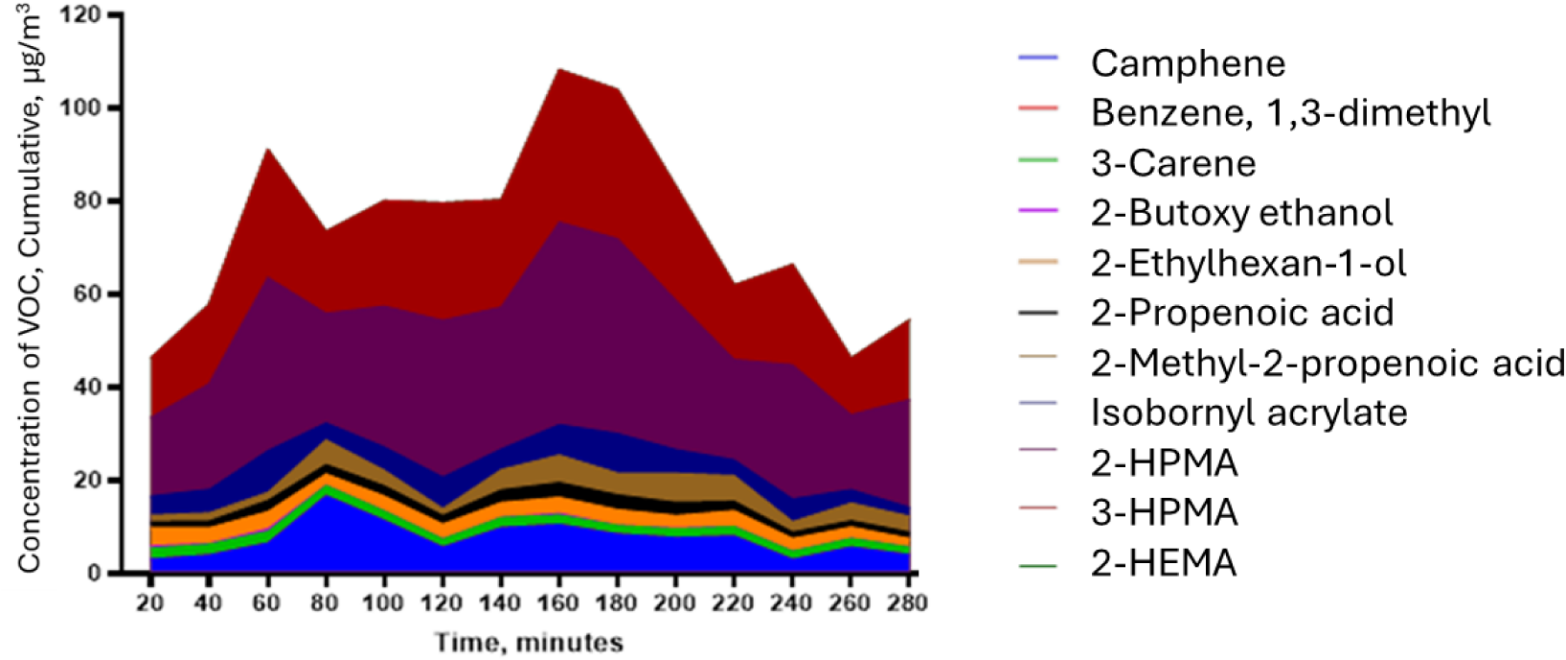
Room test scenario unenclosed: stacked VOC emissions at 50 cm from the 3D printer, without extraction, in a room. The overall height gives TVOC as a function of time.

For the room test scenario unenclosed, the 2-hydroxypropyl methacrylate concentration only reached 40 μg/m^3^; however, the cumulative effect shows that TVOC concentrations peaked at 116 μg/m^3^ at 160 min into the printing. Väisänen et al. reported TVOC ranging from 32 to 51 μg/m^3^,^44^ which is lower than this study’s findings, though they had solely considered carbonyl VOCs.

The VOC concentrations at 2 m were lower compared to the 0.5 m measurements, which shows the effect of distance as a method of exposure reduction (Figures 5 and 7). This reflected a potential scenario of sitting at a desk next to a 3D printer in a room, where increasing the distance from the source may reduce the dose and overall exposure.

**Figure 7:**
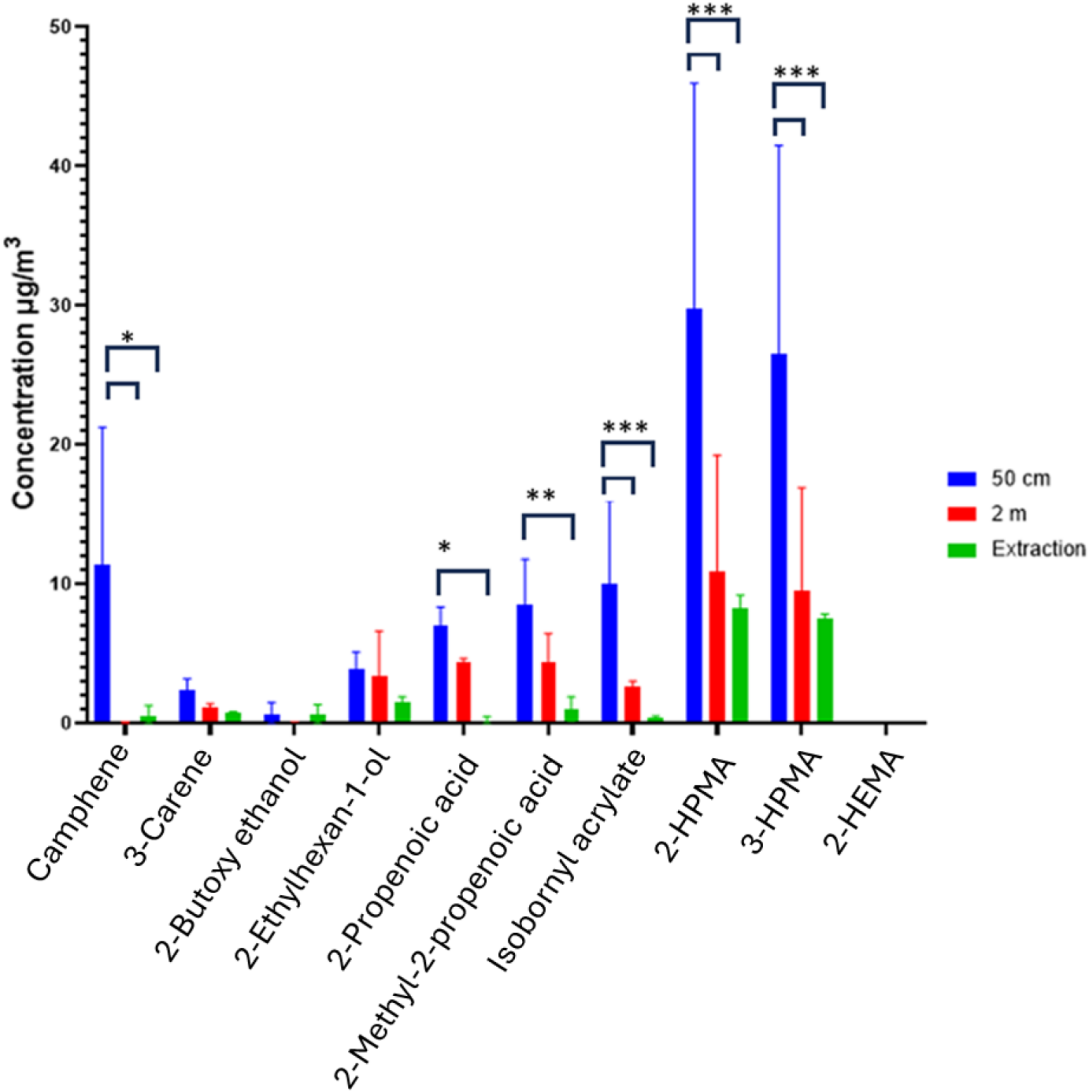
Test room scenario: the highest quantified concentration of 10 VOCs at a specific time in a room, at a distance of 50 cm, 2 m, and 50 cm with extraction. The 50 cm unenclosed values were statistically compared against the 2 m unenclosed, and against the 50 cm enclosed in the extraction hood, using a *t* test. Significance is defined as the probability of the event occurring in nature, *p*, as a decimal up to 1. The black captions on the graph refer to significant differences between the two indicated conditions, *p < 0.05, **p < 0.01, ***p < 0.001. Note—2-hydroxyethyl methacrylate has been included; however, the data points were at zero post background correction.

The effect of engineering control on reducing chemical exposure was also investigated by placing the 3D printer inside an extraction hood with samples taken 0.5 m from the 3D printer. The effectiveness of the hood can be seen in Figure 7, which results in a large decrease in VOC concentrations. The potential exposure of an operator could be reduced when sufficient extraction is employed. However, extraction systems may not be used in small businesses owing to the additional costs of buying and running an extraction hood as well as the extra space required for this equipment.

Literature on engineering control effectiveness for vat photopolymerization printers is limited. Studies of FDM printers have demonstrated that engineering controls provide substantial emission reductions: sealed enclosures with filtration achieve 80–90% VOC and UFP removal,^29^ high-flow ventilation systems achieve 95–100% reduction,^62^ and enclosures combined with local exhaust ventilation and HEPA filtration achieve 96–99% particle reduction.^63^ The VOC reductions observed in this study (71–84%) align well with these findings and demonstrate that similar control strategies are effective for resin printers.

TWA WELs were used as a comparison benchmark for the test room scenarios “unenclosed” and “enclosed”. However, it should be noted that the samplers were placed in static positions in the test room. In workplace settings, TWA measurements are based on monitoring exposure over time in the breathing zone of the worker, including periods without exposure. Individual VOC concentrations in the test room in the unenclosed and enclosed scenarios were below these WEL values^64^ by 2–3 orders of magnitude.

WELs are set to limit exposure to hazardous compounds used in workplaces in the UK. They do not apply to nonoccupational exposure in homes or “maker” communities. However, in the absence of UK-HSA air quality guidance values for most of the VOCs identified in this study, the HSE WELs were used as a benchmark to assess whether the VOC emissions reached concentrations that may cause health risks. In an occupational setting where there is a risk of harmful exposure and based on a risk assessment, control measures should be in place to mitigate these risks. These are not formal requirements for those exposed to hazards in the home or “maker” community settings but indicate whether better measures should be in place to minimize health risks.

Many quantified compounds, including 2-HEMA, 2- and 3-HPMA, and isobornyl acrylate, emitted by the printer do not have WELs. However, the total exposure to all VOC compounds ranged from ∼50 to 116 μg/m^3^. Furthermore, the concentrations of 2- and 3-HPMA and 2-HEMA from samples collected in the test chamber scenario exceeded the WELs, indicating that placing these printers in small, poorly ventilated spaces may lead to higher exposures for the operators and increase health risks.

Smaller, less ventilated rooms may be found in homes, office buildings, and hobby community centers. As the test chamber was smaller than a typical room, further dilution of the VOCs would occur, though they could pose a potentially high exposure to the occupants. Compared to the test room unenclosed and enclosed scenarios, there was a 1000-fold decrease in concentrations of the VOCs, showing the dilution effect of the room size, as well as the impact of the air exchange rate, and indicating reduced health impacts for short-duration exposures.

Different regulatory bodies publish either air quality (Environmental Agency—EA, UK Health Security Agency—UK-HSA) or WEL values (Health and Safety Executive—HSE) for specific VOCs. These can apply to various periods (15 min, 8 h, 24 h, annual averages) depending on their purpose and the circumstances. The HSE WEL values are primarily based on 8-h time-weighted average (TWA) workplace exposure limits (WELs).^64^

The guideline values from HSE, OSHA, and NIOSH for compounds structurally similar to VOCs identified in this research are given in Table 5. The concentrations of VOCs in the test room scenarios fell well below these guidance values by several orders of magnitude, indicating that the conditions in these tests and the specific resin VOC emissions were unlikely to cause adverse health effects, at least in healthy individuals.

**Table 5:** VOC Exposure Legally Enforceable Values from Health and Safety Executive (HSE) 8 h Workplace Exposure Limits, Occupational Safety and Health Administration (OSHA), and the Nonenforceable Guideline Values from the National Institute for Occupational Safety and Health (NIOSH)^a^ ^b^

	HSE, ppmv	HSE, mg m^–3^	OSHA, ppm	NIOSH, ppm
2-Hydroxypropyl acrylate	0.5	2.7		
Benzene	1	3.25	1	0.1
Formaldehyde	2	2.5	0.75	0.016
Methacrylic acid	20	72		
Methyl methacrylate	50	208		
Phenol	2	7.8	5	5
Propan-1-ol	200	500		
Propan-2-ol^b^	400	999		
Styrene	100	430	50	50
Toluene	50	191	10	100
Xylenes	50	220	100	100

a Guidelines given for the VOCs identified from this review and similar compounds as a reference for exposure limits.^64,65^ ppmv—parts per million by volume, mg m^–3^—milligrams per cubic meter. From a previous paper by these authors.^60^

b Propan-2-ol is also referenced as isopropanol in the text.

## 4 Conclusions

This study examined a commercial Formlabs Form 2 resin-based 3D printer and four resins sold in conjunction with the brand. These are examples of resins used by both businesses and home hobbyists. The tested resins were all found to emit multiple acrylic-based compounds, including 2- and 3-hydroxypropyl methacrylate, 2-hydroxyethyl methacrylate, isobornyl acrylate, and 2-propenoic acid. Many of these VOCs, including isobornyl acrylate, have not been linked to VP printing before, to the authors’ knowledge.

### 4.1 Chamber Scenario

The emission profiles were strongly dependent on resin composition, with specialized resins producing distinct chemical fingerprints related to their functional properties. The emissions from Tough, White, and Elastic Formlabs resins have not been previously examined, to the authors’ knowledge. Tough resin emitted higher concentrations of smaller cross-linking compounds like 2-hydroxyethyl methacrylate (up to 3890 μg/m^3^), while Elastic resin produced a broader range, including bulky molecules that likely contribute to flexibility. Emissions peaked at print completion when the build plate emerged from the resin bed and continued for the whole sampling period of an hour afterward, showing that the 3D printer remained a source of VOC emission even after the print cycle was completed. A recommendation for 3D printer users may be to leave the printer and printed structure within a ventilated area before returning to perform any post printing processing. During printing within the small chamber scenario, individual VOC compounds reached a peak of 68,000 μg/m^3^, with TVOC concentration exceeding 128,000 μg/m^3^ for the White resin. The chamber scenario was not representative of a realistic exposure scenario due to the small size; however, it imagines the worst-case conditions in small, poorly ventilated spaces.

### 4.2 Exposure Room Scenarios

Investigating the emission of VOCs in three separate emission scenarios gave distinct ranges of concentrations to which operators of 3D printers could be exposed. During the test room scenarios, the cumulative concentrations of all quantified VOCs adjacent to the printer ranged from 45 to 116 μg/m^3^. This represents a more realistic exposure scenario for an operator of a 3D printer, with concentrations returning to preprinting baseline within 2 h. Two practical exposure control methods demonstrated significant effectiveness: increasing the distance between the potential operator and the printer from 50 cm to 2 m (71% reduction) and using an extraction hood equipped with carbon and HEPA filters (84% reduction). These represent practical methods for exposure mitigation that can be employed in diverse settings, including homes, schools, maker spaces, and small businesses, without prohibitive costs or complex engineering solutions.

### 4.3 Health Risk Assessment and Recommendations

The primary concern is the potential health risk. As many of the compounds did not have any guidance for safety values or limits, the overall risk is unknown. This regulatory gap prevents adequate risk assessment, particularly for cumulative exposures to complex VOC mixtures. Any cumulative impact on any target within the body from total exposure to all of the identified VOCs may pose a higher potential risk than any individual VOC, even if each of the individual VOCs is below the safe guidance values.

Given the lack of evidence about the hazardous properties of many VOCs and their combined effects in total VOC emissions, a precautionary approach to minimizing these emissions is appropriate. This study presents evidence on the impact of containment, extraction, and filtration on the release of these emissions using methods relevant to the public, hobbyists, and small business users.
